# Anti-SARS-CoV-2 Antibodies in Urine of Individuals Vaccinated with Janssen AD26.COV2.S COVID-19 Vaccine

**DOI:** 10.3390/pathogens14080827

**Published:** 2025-08-21

**Authors:** Marina F. N. Melo, Rômulo C. D. Lira, Raquel S. B. Câmara, Isabela A. G. Pereira, Fernanda F. Ramos, Carolina S. F. Costa, Laura F. Amorim, Quezia D. Teixeira, Flávio G. da Fonseca, Vandack Nobre, Flavia G. F. Ferreira, Jorge Pinto, Eduardo A. F. Coelho, Fernanda Ludolf, Júlia F. M. Caporali

**Affiliations:** 1 Programa de Pós-Graduação em Ciências da Saúde, Infectologia e Medicina Tropical, Faculdade de Medicina, Universidade Federal de Minas Gerais, Belo Horizonte 30.130-100, Minas Gerais, Brazil; marina.melo.1@hotmail.com (M.F.N.M.); cdelglira@hotmail.com (R.C.D.L.); raquelsoares.id@gmail.com (R.S.B.C.); amorim.gpereira@gmail.com (I.A.G.P.); fe.fonsecaramos@gmail.com (F.F.R.); carolflorcosta@gmail.com (C.S.F.C.); laurafernamorim@gmail.com (L.F.A.); vandack@gmail.com (V.N.); eduardoferrazcoelho@yahoo.com.br (E.A.F.C.); 2 Programa de Pós-Graduação em Ciências da Saúde, Faculdade de Ciências Médicas de Minas Gerais, Belo Horizonte 30.130-110, Minas Gerais, Brazil; quezia_diast92@yahoo.com.br; 3 Laboratório de Virologia Básica e Aplicada, Departamento de Microbiologia, Instituto de Ciências Biológicas, Universidade Federal de Minas Gerais, Belo Horizonte 31.270-901, Minas Gerais, Brazil; fdafonseca@icb.ufmg.br; 4 Departamento de Pediatria, Faculdade de Medicina, Universidade Federal de Minas Gerais, Belo Horizonte 30.130-100, Minas Gerais, Brazil; ffaleiroferreira@gmail.com (F.G.F.F.); jorgeandradepinto@gmail.com (J.P.)

**Keywords:** SARS-CoV-2, COVID-19, ELISA, urine, immunoassay, AD26.COV2.S

## Abstract

Urine-based immunoassay is a non-invasive method with demonstrated utility in detecting anti-SARS-CoV-2 antibodies in unvaccinated patients with COVID-19. To evaluate urine’s potential for serological surveys in a real-world setting, SARS-CoV-2 serology was performed on urine samples from vaccinated individuals, both with and without prior confirmed COVID-19. (1) Methods: An in-house indirect ELISA was used to measure antibodies against recombinant spike (S) and nucleocapsid (N) proteins of SARS-CoV-2 in urine and paired serum from 149 individuals vaccinated with Janssen AD26.COV2.S, an S protein-based COVID-19 vaccine. (2) Results: Anti-S and anti-N levels were higher in the urine and serum of participants with confirmed prior COVID-19 compared to those without prior infection. Urinary anti-S effectively distinguished vaccinated individuals with (AUC = 0.96) and without (AUC = 0.88) prior infection from negative controls (non-vaccinated, non-previously infected individuals) (*p* < 0.0001). Among vaccinated participants, urinary anti-S and anti-N identified prior infection, with AUC values of 0.73 (*p* < 0.0001) and 0.60 (*p* = 0.03), respectively, being recorded. (3) Conclusions: Findings indicate that urinary anti-SARS-CoV-2 antibodies reflect AD26.COV2.S vaccination and previous COVID-19. To further advance the methodology, studies with larger sample sizes and a greater diversity of COVID-19 vaccines are required.

## 1. Introduction

Studies have shown that diagnosing infectious diseases by detecting antibodies in urine is a viable, safe, convenient, and non-invasive technique. This diagnostic approach has been explored for diseases such as filariasis [1], hepatitis A and C [2], schistosomiasis [3], dengue [4], strongyloidiasis [5], *Helicobacter pylori* infection [6], leishmaniasis [7,8], among others [9]. The concentration of antibodies in urine is approximately 4000 to 10,000 times lower than in serum, as the high molecular weight of immunoglobulins limits their passage through the glomerular filtration membrane in healthy kidneys. Additionally, urinary antibody concentrations vary according to an individual’s hydration status [9,10,11]. Nevertheless, antibodies can be detected in urine using the ELISA method [1], which offers several potential applications, including its use in epidemiological prevalence studies [9].

Given the advantages of urine as a serological specimen, a study conducted during the coronavirus disease 2019 (COVID-19) pandemic employed an in-house urine-based ELISA using a recombinant nucleocapsid (N) protein of severe acute respiratory syndrome coronavirus 2 (SARS-CoV-2), expressed in a prokaryotic system, to detect anti-SARS-CoV-2 antibodies in the urine of unvaccinated, hospitalized patients with qRT-PCR-confirmed infection [12]. A second study used a recombinant S protein, expressed in both eukaryotic and prokaryotic systems, to detect antibodies in unvaccinated, hospitalized patients with acute COVID-19 [13]. A third validation study successfully detected antibodies against partial chimeric SARS-CoV-2 spike (S) and N proteins (expressed in a prokaryotic system) in urine samples from hospitalized patients with acute COVID-19, highlighting the method’s convenience and cost-effectiveness compared to serum-based assays, especially in settings where sample collection is challenging [14].

With the introduction of COVID-19 vaccines, it was hypothesized that urinary anti-N antibodies could serve as markers of prior infection in individuals vaccinated exclusively with spike protein-based vaccines [15]. However, the chimeric protein did not detect antibodies in a small group of individuals vaccinated with the Janssen Ad26.COV2.S COVID-19 vaccine [14]. The presence and persistence of urinary SARS-CoV-2 antibodies in vaccinated individuals several months after vaccination or acute disease remains to be further explored.

To address this knowledge gap, we conducted an exploratory study to evaluate urinary levels of anti-N and anti-S antibodies in adults vaccinated with the Janssen Ad26.COV2.S COVID-19 vaccine, which employs the spike (S) protein as its sole antigen. For analysis, participants were stratified into groups based on whether or not they had a prior history of COVID-19 confirmed by RT-PCR.

## 2. Materials and Methods

### 2.1. Study Design

This comparative, cross-sectional, observational exploratory study analyzed anti-S and anti-N antibodies in urine and serum samples from individuals vaccinated with the Janssen Ad26.COV2.S COVID-19 vaccine.

### 2.2. Study Population

#### 2.2.1. Vaccinated Individuals

Eligible participants were adults aged 18 years or older, in good health, residing in Belo Horizonte, Minas Gerais, Brazil, who had received the Janssen Ad26.COV2.S COVID-19 vaccine between November 2020 and December 2021. Individuals who had received any other COVID-19 vaccines were excluded. Serum and urine samples were collected between May and July 2022.

Between November 2020 and July 2022, all participants presenting with suspected COVID-19 symptoms underwent nasopharyngeal swab collection for SARS-CoV-2 RT-PCR testing. A prior COVID-19 diagnosis was confirmed by a positive RT-PCR result.

Vaccinated individuals without prior RT-PCR-confirmed COVID-19 were classified as Group 1 (G1), while those with a confirmed prior infection were classified as Group 2 (G2).

#### 2.2.2. Unvaccinated Individuals

Unpaired urine and serum samples collected from individuals before the pandemic, or from unvaccinated individuals who adhered to strict quarantine and remained asymptomatic during the pandemic, were used as a negative comparison group. These individuals were considered to have never been infected with SARS-CoV-2. These individuals comprised Group 0 (G0).

### 2.3. Sample Collection

Blood (20 mL) and urine (80 mL) paired samples were collected concurrently from each participant. Urine samples were collected at any time of day and without a specific urine retention time, although collecting urine in the morning or after an average retention period of 4 h was recommended when feasible. Urine samples were collected in sterile 80 mL bottles and then transferred to 15 mL tubes containing sodium azide (71289, Sigma-Aldrich^®^, St. Louis, MO, USA) at a final concentration of 0.1% (*v*/*v*). The tubes were transported at room temperature and stored at 2 to 8 °C until use. Pre-pandemic urine samples had been collected before 2019 and kept refrigerated (2 to 8 °C) prior to use.

Blood samples were obtained via venipuncture using sterile 20 mL collection tubes containing serum separator gel but no anticoagulant. The tubes were centrifuged at 3500 rpm for 15 min at 4 °C. The serum was then collected and stored in conical Eppendorf tubes at −20 °C until use.

### 2.4. ELISA

The ELISA method was applied following established protocols from prior studies [12], adhering to optimal experimental conditions for each recombinant SARS-CoV-2 protein type—prokaryotic N (FAPON, Dongguan, China, catalog number 516) [12], eukaryotic S (FAPON, China, catalog number 537), and in-house prokaryotic S [13,16]—and for both urine and serum samples.

Polystyrene plates (high-binding 96-well polystyrene microplate—Corning, Merck, Darmstadt, Germany) were coated with recombinant proteins diluted in carbonate buffer for 18 h at 4 °C, with 400 ng of antigen/well of each recombinant N and S protein (prokaryotic and eukaryotic), as previously established by Ludolf [12], Ramos [13] and Bagno [16]. After sensitization, blocking was performed using 200 µL of a solution containing 1× PBS, 0.05% Tween 20 (PBS-T), and 1% BSA for 2 h at 37 °C. The plates were then washed 5 times using PBS-T and incubated with 100 µL of undiluted urine for 1 h at 37 °C. The plates were washed five times using PBS-T and then incubated with 100 µL of peroxidase-conjugated human anti-IgG antibody at a 1:10,000 dilution for N commercial, S commercial, and S in-house proteins in PBS-T for 1 h at 37 °C. Again, the plates were washed 5 times with PBS-T and the reactions were developed using TMB chromogenic solution (3,3′,5,5; tetramethylbenzidine, Moss, Franklin Park, IL, USA) for 30 min in the dark. The reaction was stopped by adding H_2_SO_4_ (0.5 M) and the optical density (OD) was read in a spectrophotometer for ELISA microplates (Multiskan Go, Thermo Scientific, Waltham, MA, USA) at λ450 nm.

The serum assays followed a previously optimized protocol [12,16]. The sensitization of the plates was performed using 400 ng of each of the recombinant commercial proteins N and S, and in-house S protein with serum dilution (1:100) and anti-IgG antibody (FAPON, China) 1:40,000 for N and 1:10,000 for S, with incubation times of 30 min each.

### 2.5. Statistical Analyses

This was an exploratory study with a convenience sample size of at least 20 participants per group. Data were analyzed using GraphPad Prism (version 8.0 for Windows) and SPSS (version 15.0 for Windows). Continuous variables were presented as mean ± standard deviation or median and interquartile range, as appropriate. Categorical variables were expressed as proportions. Comparisons of continuous variables between groups were made using Student’s *t*-test, and proportions were compared using the chi-square test. Pearson’s correlation coefficient was used to assess relationships between continuous variables. Receiver operating characteristic (ROC) curves were generated to evaluate sensitivity and specificity. A *p*-value < 0.05 was considered statistically significant.

## 3. Results

### 3.1. Baseline Characteristics

The study included 149 individuals vaccinated with the Ad26.COV2.S vaccine, of whom 71 (48%) had a history of COVID-19 confirmed by RT-PCR. None of the participants had been hospitalized due to the infection.

Among the 78 vaccinated participants without confirmed COVID-19 (Group 1), 51% were male, compared to 52% in the group of 71 vaccinated individuals with a history of infection (Group 2) (*p* = 0.919). A significant difference in age was observed: the mean age in Group 1 was 58 years (±12), while in Group 2 it was 51 years (±13) (*p* = 0.001). Regarding numbers of vaccine doses, two participants in each group had received three doses, while the remaining participants had received two doses. This resulted in a mean number of doses of 2.03 (±0.159) for Group 1 and 2.03 (±0.167) for Group 2 (*p* = 0.925). There was no significant difference in the time elapsed between the last vaccine dose and sample collection: the mean interval was 5.77 months (±0.81) for Group 1 and 5.87 months (±0.41) for Group 2 (*p* = 0.329). In Group 2, the median time between COVID-19 diagnosis and sample collection was 138 days [IQR: 119–160].

The negative control group (Group 0) consisted of 23 unpaired urine samples and 24 serum samples collected either before the pandemic or from unvaccinated individuals who maintained strict quarantine and remained asymptomatic during the pandemic, and were therefore considered to have never been infected with SARS-CoV-2. Among the individuals from whom urine samples were obtained, 43% were female, 22% male, and 35% had unknown sex due to the use of a biobank. Age data were available for 50% of these individuals, and a mean age of 28.25 years (±8.99) was recorded. For the serum samples, 54% were from females, 13% from males, and 33% had unknown sex. The mean age was 32.8 years (±10.57), with age data being unavailable for 33% of the individuals. Compared to Groups 1 and 2, Group 0 had a significantly higher proportion of females and a lower mean age.

### 3.2. Anti-SARS-CoV-2 Antibodies Results in Urine and Serum

**Figure 1:**
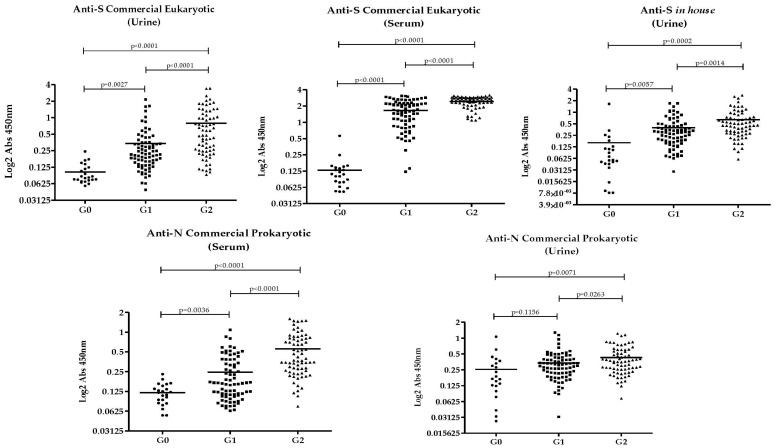
Comparison of antibody detection against each recombinant protein, in urine and serum, among groups. G0: unvaccinated without prior infection. G1: vaccinated without prior infection. G2: vaccinated with prior infection.

The detection of antibodies in urine and serum using different recombinant SARS-CoV-2 proteins is illustrated in Figure 1. Overall, antibody levels against all studied proteins were highest in Group 2, followed by Group 1, and lowest in Group 0. This trend was consistent for both urine and serum samples. The only instance where no statistically significant difference was observed between groups was in the detection of anti-N antibodies in urine when comparing Group 0 and Group 1.

### 3.3. Influence of Age, Sex, and Urine Retention Time on Antibody Levels

In the intragroup analysis, no correlation was observed between age or sex and antibody levels in either serum or urine. Urine retention time prior to sample collection was recorded for 60% of participants in Group 1 and 56% in Group 2, with no significant difference between the groups (150 ± 97 min vs. 125 ± 86 min; *p* = 0.203). Urine retention time did not correlate with antibody levels in either group. All participants were assessed by a physician at the time of collection and were considered clinically well-hydrated.

### 3.4. Correlation Between Antibody Responses to Recombinant SARS-CoV-2 Proteins

In Group 1 (G1), a direct correlation was observed between anti-S antibody levels in serum and urine for the commercial spike protein (r = 0.522, *p* < 0.001). In Group 2 (G2), antibody levels showed significant positive correlations between serum and urine for both the nucleocapsid (N) protein (r = 0.311, *p* = 0.008) and the commercial spike (S) protein (r = 0.378, *p* = 0.001). Additionally, in G2, a direct correlation was observed between anti-N and anti-S antibody levels within the same biological matrix: in serum (r = 0.352, *p* = 0.003) and in urine (r = 0.488, *p* < 0.001). Urinary anti-S levels also showed strong correlations between the commercial and in-house spike proteins in both G1 (r = 0.460, *p* < 0.001) and G2 (r = 0.723, *p* < 0.001).

### 3.5. Accuracy of Recombinant SARS-CoV-2 Proteins

The accuracy of each rSARS-CoV-2 protein was assessed in urine and serum. The area under the curve (AUC), sensitivity, and specificity values are presented in Table 1. Receiver operating characteristic (ROC) curves were generated to distinguish the following: (i) negative controls (unvaccinated and uninfected individuals, G0) from vaccinated individuals with confirmed prior infection (G2); (ii) negative controls from vaccinated individuals without confirmed prior infection (G1); and (iii) vaccinated individuals with confirmed prior infection (G2) from those without confirmed prior infection (G1). Overall, the proteins showed the highest diagnostic accuracy when used to differentiate G0 from G2.

## 4. Discussion

Our findings demonstrate that antibodies against recombinant S and N proteins of SARS-CoV-2 are detectable in the urine of individuals vaccinated with the Janssen Ad26.COV2.S COVID-19 vaccine, with higher levels observed in those with a confirmed prior infection (Group 2). Notably, none of the participants with prior confirmed infection required hospitalization, and the median time since the last COVID-19 episode was 138 days [IQR: 119–160]. The average time since the last vaccine dose was 5.77 (±0.81) months for Group 1 and 5.87 (±0.41) months for Group 2 (*p* = 0.329). These results suggest that urinary anti-N and anti-S antibodies can persist for several months following an immunizing event, whether through isolated spike-based vaccination or mild-to-moderate natural infection.

These are relevant findings, as previous studies evaluating urinary anti-N and anti-S antibodies focused exclusively on samples collected during the acute phase of COVID-19 [12,13]. Additionally, testing for the presence of these antibodies in the urine of vaccinated individuals had previously been conducted using a very small sample, and no significant detection was recorded—possibly due to the nature of that specific chimeric protein or the limited sample size [14]. Given that immunoglobulin levels in urine are thousands of times lower than in serum [9], detecting anti-N and anti-S antibodies in urine five to six months after vaccination or infection suggests that the method has good sensitivity. This is particularly noteworthy considering that serum anti-N and anti-S antibody peaks typically occur around one month after natural infection [17], and anti-S peaks approximately two months after Janssen vaccination [18], with significant declines noted after five and eight months, respectively.

When comparing unvaccinated, uninfected individuals (Group 0) with vaccinated individuals who had had a prior infection (Group 2), we observed significantly higher reactivity to all tested proteins in both urine and serum. This reflects elevated anti-S levels due to combined immune stimulation from vaccination and infection. In contrast, the presence of anti-N antibodies specifically indicates a humoral response to infection, as the Ad26.COV2.S vaccine does not include the nucleocapsid protein.

When comparing vaccinated individuals without confirmed prior infection (Group 1) to unvaccinated, uninfected controls (Group 0), higher urinary and serum anti-S antibody levels were observed in Group 1, confirming an immune response to vaccination. This aligns with previous studies showing the efficacy of the Ad26.COV2.S vaccine against severe and critical COVID-19 [18,19], as well as its association with elevated neutralizing antibody levels [20].

As expected, anti-N protein levels in urine were lower in individuals without confirmed prior infection, regardless of vaccination status. However, vaccinated individuals without a confirmed infection (Group 1) showed higher anti-N levels in serum—and a similar trend in urine—when compared to unvaccinated controls. This may suggest prior undiagnosed asymptomatic SARS-CoV-2 infections in Group 1, because quarantine was not mandatory in this group. Indeed, asymptomatic infections have been reported in approximately 25% of the general population [21]. Although this limits the validity of Group 1 as a strictly negative comparator for prior infection, it also highlights the potential utility of urinary anti-N detection for identifying past asymptomatic infections in individuals vaccinated exclusively with spike-based vaccines.

Another notable finding was the significantly higher urinary anti-S reactivity observed in vaccinated individuals with prior infection compared to those without, with the following results being obtained: for the eukaryotic S protein, G1 (0.3397 ± 0.04176) vs. G2 (0.7876 ± 0.08827), *p* < 0.0001; for the in-house S protein, G1 (0.3931 ± 0.03978) vs. G2 (0.6386 ± 0.06637), *p* < 0.0001. This is likely due to hybrid immunity—the combined effect of infection and vaccination—which has been shown to elicit stronger immune responses than either vaccination or infection alone [22].

Regarding diagnostic accuracy, urinary anti-S antibody detection showed excellent performance in distinguishing unvaccinated, uninfected individuals (G0) from vaccinated, previously infected ones (G2) using both the commercial and in-house spike proteins (AUC = 0.96 and 0.92; *p* < 0.0001). It also demonstrated very good accuracy for distinguishing G0 from G1 (AUC = 0.88 and 0.85; *p* < 0.0001). Differentiating G1 from G2 showed moderate-to-good performance [AUC = 0.67 (commercial S) and 0.73 (in-house S); *p* < 0.001], which likely reflects the stronger anti-S response associated with hybrid immunity.

On the other hand, the accuracy of urinary anti-N in identifying prior infection among vaccinated individuals was lower (AUC = 0.603, *p* = 0.03), likely due to a proportion of undiagnosed asymptomatic infections in Group 1. Urinary anti-N performed better when comparing G0 and G2 (AUC = 0.74, *p* < 0.001). The higher accuracy in a previous study [12] is possibly explained by the shorter interval between infection and sample collection (14–21 days), which coincides with the anti-N antibody peak at 3–5 weeks post-infection [17].

Antibody levels against the tested proteins showed moderate-to-strong intragroup correlations between serum and urine, with the strongest correlation observed for the commercial spike protein (r = 0.522, *p* < 0.001). A strong correlation was not necessarily expected, as urinary antibody levels depend not only on serum concentrations but also on renal filtration and urine concentration [9]. In Group 2, moderate correlations were also observed between anti-N and anti-S responses in both serum and urine, potentially reflecting infection-induced enhancement of anti-S responses [22]. Furthermore, urinary anti-S levels showed good correlation between the commercial and in-house spike proteins, supporting the reliability of the in-house prokaryotic S protein.

No association was observed between age and antibody levels, possibly due to the limited sample size or absence of age extremes. Although aging is associated with decreased immune responses (immunosenescence) [23], Group 1—despite being on average seven years older than Group 2—still had higher anti-S and anti-N levels than Group 0, whose participants were considerably younger. Regarding sex, the literature indicates that females mount stronger humoral responses than males [24]. As Groups 1 and 2 had similar sex distributions, comparisons between them are reliable. However, Group 0 included a higher proportion of younger females (median age 26–29), which may have raised the negative cutoff threshold. If demographic characteristics had been balanced across groups, the antibody differences between G0 and the vaccinated groups would likely have been even more pronounced, while differences between G1 and G2 might have been reduced.

This exploratory study has several limitations. First, quantitative comparisons between samples should be interpreted with caution, as results were not adjusted for urine concentration. While urinary antibody levels may fluctuate due to hydration status, prior studies on Wuchereria bancrofti and *H. pylori* found only modest effects from retention time or urine dilution on ELISA performance [1,9]. Other factors such as pH and microbial contamination can influence antibody detection; however, these effects were minimized by the addition of sodium azide. Significant proteinuria, however, may still affect assay results. Ludolf et al. [12] emphasized that urine-based assays are reliable for qualitative purposes, although their quantitative interpretation remains limited. Their findings also support the feasibility of using samples collected at various times of day. Specifically, in our study, all participants were assessed by a physician at the time of collection and were considered clinically well-hydrated. Although such assessment could not be considered a substitute for serum creatinine or urine concentration measurements, it did indicate that no cases of overt dehydration were present. Urine retention time prior to sample collection showed no significant difference between the G1 and G2 groups, and did not correlate with antibody levels in either group. We assume that urine concentration, although a potential confounding factor, was likely balanced between groups. Future studies should aim to normalize urinary antibody levels against parameters such as creatinine, total protein, and urine concentration.

Second, the limited sample size precluded analysis by time since vaccination or infection—an important variable, as antibody titers decline over time [17,18,19,20,21,22]. Additionally, the absence of an unvaccinated group with prior infection restricted our ability to compare pure post-infection immunity with hybrid immunity. Lastly, the exclusive focus on recipients of the Janssen Ad26.COV2.S vaccine limits the generalizability of our findings to individuals vaccinated with other COVID-19 vaccines.

Despite these limitations, this study serves as a proof-of-concept which demonstrates that an immunoassay can detect anti-S and anti-N antibodies in urine several months after Ad26.COV2.S vaccination and mild-to-moderate SARS-CoV-2 infection. These results contribute to a growing body of evidence [12,13,14] and support the further development and validation of this methodology in larger studies.

Although this method is not currently suited for COVID-19 diagnosis—due to widespread seropositivity from vaccination and prior infections—urine-based immunoassays may represent a safe, convenient alternative for initial clinical and epidemiological assessment in future outbreaks.

## Figures and Tables

**Table 1:** Accuracy of each rSARS-CoV-2 protein in urine and serum for distinguishing between groups. G0: unvaccinated non-previously infected. G1: vaccinated without confirmed prior infection. G2: vaccinated with confirmed prior infection. In bold: accuracy with AUC > 0.90.

			Sensibility	Specificity	AUC	*p*-Value
**Anti-N Commercial Prokaryotic**	**serum**	**G0 × G2**	**91.55**	**91.3**	**0.9547**	**<0.0001**
	serum	G0 × G1	61.54	60.87	0.7018	0.0034
	serum	G2 × G1	73.24	69.23	0.7879	<0.0001
	urine	G0 × G2	77.46	61.9	0.7428	0.00076
	urine	G0 × G1	67.95	61.9	0.6716	0.016
	urine	G2 × G1	60.56	50	0.603	0.03
**Anti-S Commercial Eukaryotic**	**serum**	**G0 × G2**	**98.59**	**100**	**0.9988**	**<0.0001**
	**serum**	**G0 × G1**	**97.44**	**95.65**	**0.9875**	**<0.0001**
	serum	G2 × G1	77.14	70.51	0.7866	<0.0001
	**urine**	**G0 × G2**	**88.73**	**91.3**	**0.9602**	**<0.0001**
	urine	G0 × G1	83.33	78.26	0.8824	<0.0001
	urine	G2 × G1	69.01	62.82	0.7294	<0.0001
**Anti-S in-house Prokaryotic**	**urine**	**G0 × G2**	**90**	**82.61**	**0.9155**	**<0.0001**
	urine	G0 × G1	83.33	78.26	0.8478	<0.0001
	urine	G2 × G1	65.71	60.26	0.6737	0.00027

## Data Availability

The original contributions presented in this study are included in the article. Further inquiries can be directed to the corresponding authors.
